# Identification of the First* De Novo UBIAD1* Gene Mutation Associated with Schnyder Corneal Dystrophy

**DOI:** 10.1155/2016/1968493

**Published:** 2016-06-12

**Authors:** Benjamin R. Lin, Ricardo F. Frausto, Rosalind C. Vo, Stephan Y. Chiu, Judy L. Chen, Anthony J. Aldave

**Affiliations:** Stein Eye Institute, David Geffen School of Medicine at UCLA, 100 Stein Plaza, Los Angeles, CA 90095-7003, USA

## Abstract

*Purpose.* To report the identification of the first* de novo UBIAD1* missense mutation in an individual with Schnyder corneal dystrophy (SCD).* Methods.* A slit lamp examination was performed on a 47-year-old woman without a family history of corneal disorders. The proband's parents, two sisters, and son were also examined and genomic DNA from all six individuals was collected. The exons and exon-intron boundaries of* UBIAD1* were screened using Sanger sequencing. Identified mutations were screened for in 200 control chromosomes.* In silico* analysis predicted the impact of identified mutations on protein function and structure.* Results.* Slit lamp examination of the proband revealed findings consistent with SCD. Corneas of the family members appeared unaffected. Screening of* UBIAD1* in the proband identified a novel heterozygous c.308C>T mutation, predicted to encode the missense amino acid substitution p.(Thr103Ile). This mutation was not identified in any of the family members or in 200 control chromosomes and was predicted to be damaging to normal protein function and structure.* Conclusions.* We present a novel heterozygous* de novo* missense mutation in* UBIAD1*, p.(Thr103Ile), identified in a patient with classic clinical features of SCD. This highlights the value of genetic testing in clinical diagnostic settings, even in the absence of a positive family history.

## 1. Introduction

Schnyder corneal dystrophy (SCD; MIM #21800) was first described by Van Went and Wibaut in 1924 and later by Schnyder in 1929 [1, 2]. Formerly known as Schnyder crystalline corneal dystrophy or SCCD, it was subsequently renamed in 2008 by the International Committee for the Classification of Corneal Dystrophies to SCD, as only about half of affected patients demonstrate corneal crystals on examination [3–5]. SCD is a rare autosomal dominant disorder associated with the development of central corneal stromal opacification with or without subepithelial or anterior stromal crystalline deposition in the first or second decade of life [4, 6, 7]. Subsequently, affected individuals develop bilateral arcus lipoides and progressive stromal opacification [4, 6, 7].

While SCD has been associated with systemic hyperlipidemia, individuals with SCD have both normal and abnormal serum lipid, lipoprotein, and cholesterol levels, and Lisch and colleagues have found a lack of correlation between serum lipid levels and corneal findings [6, 8–14]. Nevertheless, a localized (i.e., corneal) dysregulation of lipid/cholesterol transport or aberrant lipid metabolism is thought to be a possible molecular cause of the SCD phenotype [6, 15]. In 1996, Shearman et al. performed genome-wide linkage analysis on two Scandinavian families with SCD and demonstrated linkage to a locus on chromosome 1 (1p.34.1–36) [16]. Eleven years later, Orr and colleagues and Weiss and colleagues independently identified mutations in several highly conserved regions of the* UbiA prenyltransferase domain containing 1* (*UBIAD1*) gene, located on chromosome 1p36 in eleven families [15, 17]. To date, a total of 25 mutations have been reported, but none were demonstrated to be spontaneous (Table 1). Herein, we report the first confirmed* de novo* mutation associated with SCD. As such, we recommend that molecular genetic analysis be considered to confirm or refute a suspected clinical diagnosis of SCD, even in the setting of a negative family history.

## 2. Materials and Methods

Informed consent was obtained from all subjects according to the tenets of the Declaration of Helsinki. The Institutional Review Board at The University of California, Los Angeles, approved the study described herein (UCLA IRB # 11-000020).

### 2.1. Slit Lamp Imaging, DNA Collection, and Preparation

**Figure 1:**
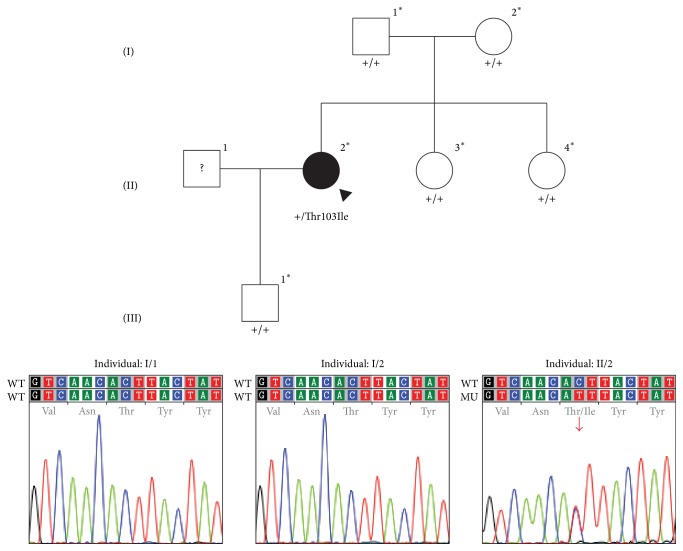
Pedigree of family with Schnyder corneal dystrophy. Filled symbols represent affected individuals and unfilled symbols represent unaffected individuals. Question marks indicate individuals of unknown affected status. The proband is designated with a black arrowhead. Asterisks indicate individuals in whom* UBIAD1* screening was performed; below these symbols, the results are given as wild type (+) or the identified mutation is shown. Chromatograms demonstrate the results of sequencing* UBIAD1* in the parents of the proband and the proband, in whom the identified heterozygous c.308C>T missense mutation is indicated with a red arrow.

Slit lamp biomicroscopic imaging was performed for the proband and five family members to determine their affected status (Figure 1). After obtaining informed consent, saliva samples were collected from each using the Oragene Saliva Collection Kit (DNA Genotek, Inc., Ontario, Canada). Genomic DNA was extracted from buccal epithelial cells using the Oragene prepIT•L2P protocol for genomic DNA purification (DNA Genotek, Inc.). In addition, genomic DNA samples previously collected from 100 healthy individuals were used as controls.

### 2.2. Polymerase Chain Reaction (PCR)

The exonic regions of* UBIAD1* were amplified using previously described primers [20]. Reactions were performed in 25 *μ*L reaction volumes containing 5 *μ*L of KAPA GC/A buffer (Kapa Biosystems, Wilmington, MA), 25 mM dNTPs, 1 unit of Kapa 2G Robust DNA polymerase (Kapa Biosystems), 60 *μ*M of each forward and reverse primer, 20–40 ng of DNA, and 17.5 *μ*L of nuclease-free H_2_O. Reactions were cycled with a denature step at 98°C for 3 min followed by 36 cycles with 98°C for 25 s, 54°C for 30 s, 72° for 30 s, and a final extension step at 72°C for 10 min. An iCycler or C1000 Touch Thermal Cycler (Bio-Rad, Hercules, CA) was used to perform thermal cycling.

### 2.3. DNA Sequencing

Prior to sequencing, the amplicon was purified by treating 15–30 ng of amplicon with 5 units of Exonuclease I and 0.5 units of Shrimp Alkaline Phosphatase (USB Corp., Cleveland, OH) followed by incubating at 37°C for 15 min and inactivating at 80°C for 15 min. Sequencing was performed in 10 *μ*L reaction volumes containing BigDye Terminator Mix v3.1 (Applied Biosystems, Foster City, CA), BDX64 enhancing buffer (MCLAB, San Francisco, CA), and 0.2 *μ*L primer (10 pmoles/*μ*L) following the manufacturer's (MCLAB) instructions. Sequences were viewed using Chromas (Technelysium, South Brisbane, Australia), and the* UBIAD1* exon and exon-intron junction sequences were compared with the NCBI reference sequence for* UBIAD1* (Genbank Accession: NM_013319.2). Identified sequence variants were annotated according to the HGVS nomenclature guidelines (http://www.hgvs.org/mutnomen/).

### 2.4. Paternity Testing

DNA samples were submitted to the University of California, Los Angeles Clinical Microarray Core, for paternity testing. The proband and her parents were genotyped for a sex associated marker, amelogenin, and 10 different short tandem repeats using the Geneprint 10 System (Promega Corporation, Madison, WI).

### 2.5. *In Silico* Protein Analysis

A webtool, DNA to protein (http://www.ebi.ac.uk/Tools/st/emboss_transeq/), was used to generate an* in silico* translation of the mutant* UBIAD1* sequence. PredictProtein was used to predict the number of polynucleotide and protein binding sites of UBIAD1 protein (https://www.predictprotein.org/). PolyPhen-2, SIFT, and PANTHER were used to predict the functional impact of identified variants [29–31]. Multiple sequence alignment of UBIAD1 orthologs from multiple species was performed using MUSCLE (http://www.ebi.ac.uk/Tools/msa/muscle/), and ConSurf (http://consurf.tau.ac.il/) was used to calculate the evolutionary conservation score for all UBIAD1 residues [32, 33]. Seven different modeling algorithms (Tmpred, PHDhtm, TMHMM 2.0, HMMTOP 2.0, MEMSAT3, MEMSAT-SVM, and TMSEG) were used to predict the transmembrane helices of both wild type and mutant forms of UBIAD1 [34–40]. TOPO2 (http://www.sacs.ucsf.edu/TOPO2/) was used to generate a consensus image from the various different transmembrane helix predictions.

## 3. Results

### 3.1. Clinical Findings

**Figure 2:**
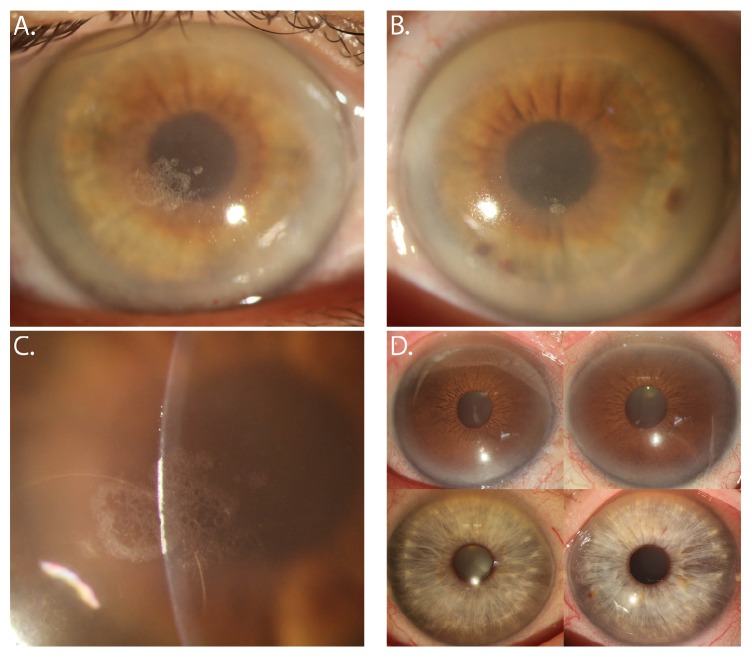
Slit lamp photomicrographs of 47-year-old woman with Schnyder corneal dystrophy. (A) Diffuse illumination of the right eye demonstrates arcus lipoides and a central crystalline deposit. (B) Diffuse illumination of the left eye also demonstrates dense arcus lipoides, central discoid haze, and a focal central crystalline deposit overlying the inferior pupillary border. (C) Slit illumination of the right eye demonstrates the subepithelial location of the crystalline deposit. (D) Slit lamp photomicrographs of the proband's father demonstrate arcus senilis but no central stromal opacification in either the right or left eye (top images). The proband's mother's corneas are clear (bottom images).

A 47-year-old woman of Japanese (father) and European (mother) descent was referred to the authors (Rosalind C. Vo and Anthony J. Aldave) for evaluation of bilateral corneal opacities associated with crystalline deposits. Her only visual complaint was that of glare, which worsened in the evening. The proband's medical history was negative for genu valgum but was significant for hyperlipidemia and hypothyroidism, for which she was taking levothyroxine. Her total cholesterol was 230 mg/dL, including a low-density lipoprotein level of 125 mg/dL and an increased high-density lipoprotein level of 90 mg/dL. Her mother also had a history of hyperlipidemia, but the patient denied a family history of a corneal disorder or decreased vision. Corrected Snellen visual acuities measured 20/20- in both eyes. Slit lamp biomicroscopic examination demonstrated bilateral arcus lipoides and central corneal, discoid, and pan-stromal opacification (Figure 2). Superficial crystalline deposits were observed in each cornea, right eye more than left. Neither parent demonstrated crystalline corneal changes or central or peripheral corneal opacification (Figure 2). Similarly, neither of the proband's siblings nor her son demonstrated any corneal opacities. Out of the 100 control individuals, 26 individuals were of Asian descent, and 74 were of Caucasian descent. In addition, 39 were male and 60 were female. No corneal opacities were seen in any of the 100 control individuals.

### 3.2. Genetic Screening

Both coding exons of* UBIAD1* were screened in the proband. A novel heterozygous variant (c.308C>T), which is predicted to encode a missense p.(Thr103Ile) amino acid change, was identified in exon 1 (Figure 1). This variant was not identified in either of the proband's parents or in her siblings or offspring (Figure 1). In addition, it was not identified in 200 control chromosomes nor reported in dbSNP 142 or the 1000 Genomes database, which references Ensembl 76. Genotyping of the proband and each of her parents confirmed (with greater than 99.999% certainty) that the proband's mother and father were her biological parents (Table 2) [41].

### 3.3. Protein Bioinformatics Analysis

**Figure 3:**
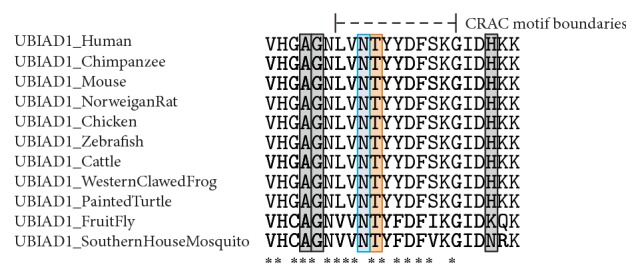
MUSCLE alignment of 11 different UBIAD1 homologues showing binding motifs. Shown are excerpts of homologous sequences of 11 different species, corresponding to amino acid residues 93 to 115 in human UBIAD1 protein. The boundaries of the cholesterol recognition/interaction amino acid consensus (CRAC) sequence are denoted by the dashed lines. The Asn102 and Thr103 residues are highlighted in blue and orange, respectively. All other reported mutations in this region associated with SCD are highlighted in gray. An asterisk (*∗*) indicates residues that are fully conserved across all 11 aligned homologues.

**Figure 4:**
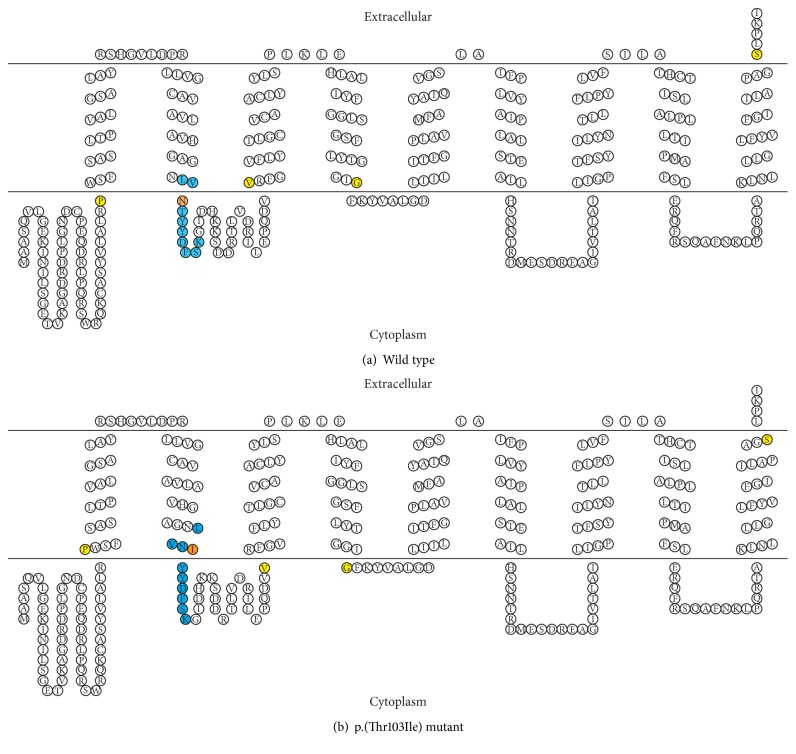
Consensus model of the wild type and mutant UBIAD1 protein. This shows the consensus figure from seven different modeling algorithms. (a) Wild type UBIAD1 protein. (b) UBIAD1 protein with a p.(Thr103Ile) substitution. The CRAC binding domain is shown in blue, and amino acid 103 is shown in orange. All other amino acids affected by structural changes to transmembrane helices caused by the p.(Thr103Ile) substitution are shown in yellow. Note that the folds seen in the diagram do not represent alpha-helices or beta sheets. Rather, they indicate residue location in relation to the cell membrane.

By* in silico* analysis, the UBIAD1 protein is predicted to have seven protein binding sites and one polynucleotide binding site, none of which include amino acid 103. However, this amino acid is within a conserved casein kinase II phosphorylation site, as well as a cholesterol recognition/interaction amino acid consensus (CRAC) sequence. PolyPhen-2, SIFT, and PANTHER all predict the p.(Thr103Ile) substitution to be likely damaging to protein function. ConSurf calculations assigned amino acid 103 a score of 9, which indicates maximal conservation across species. This is supported by MUSCLE alignment of sequences from 11 different species (Figure 3). The seven different modeling algorithms generated structural models with some resemblance to one another. While the various models did not perfectly agree, there was sufficient similarity between all of them to generate a consensus model for wild type UBIAD1 protein based on the transmembrane start and stop predictions (Figure 4(a)). Because isoleucine, a highly hydrophobic amino acid, replaces threonine, a polar/hydrophilic amino acid, the p.(Thr103Ile) substitution is predicted to extend the length of the second transmembrane helix to include an additional two residues. This is predicted to move amino acid residues 102 and 103 from their normal extracellular location to being embedded within the plasma membrane (Figure 4(b)).

## 4. Discussion

Schnyder corneal dystrophy (SCD) has been associated with 25 different mutations in* UBIAD1* in the 54 families in which screening has been reported to date. One mutation, p.(Asn102Ser), has been identified in 20 of these 54 families, which are of Swede-Finn, American, British, Italian, German, Irish, Czech, Chinese-Taiwanese, Japanese, and Polish descent [15, 17, 18, 20–23]. Given the disparate ancestry of the families in which the p.(Asn102Ser) mutation has been identified, the Asn102 residue is considered a hotspot for SCD and likely arose spontaneously in each population in which it has been identified [21]. Herein, we report a proband in whom the p.(Thr103Ile) mutation involving the adjacent amino acid has been confirmed to have arisen spontaneously. This makes* UBIAD1* only the third gene in which spontaneous mutations have been confirmed to be associated with a corneal dystrophy, in addition to* ZEB1* mutations associated with posterior polymorphous corneal dystrophy 3 [42, 43] and* TGFBI* mutations associated with Reis-Bücklers corneal dystrophy [44, 45], Thiel–Behnke corneal dystrophy [45], granular corneal dystrophy type 2 [46], and lattice corneal dystrophy [47].

Previously published work established that UBIAD1 primarily acts as a mitochondrially localized prenyltransferase and has an active domain from residues 58–333, in which all reported mutations are located [17, 18, 21]. Previous computational modeling has predicted that the Asn102 residue plays a key role in identifying aromatic substrates for prenylation, as well as recognition and stabilization of a variety of other substrates [18]. Work done by Huang in a human UBIAD1 homologue, AfUbiA from* Archaeoglobus fulgidus*, suggests that the Asn102 residue in UBIAD1 (homologue to Asn68 in AfUbiA) is likely to play a critical role in protein function, acting as part of an Mg^2+^/diphosphate binding site that stabilizes the diphosphate on the prenyl donor substrate [48]. The Asn102 residue is reported to be at the boundary of a transmembrane helix, although various modeling algorithms differ in reporting its exact location with respect to the transmembrane region [18, 21, 27, 49].

We propose two potential mechanisms via which the p.(Thr103Ile) mutation, located adjacent to Asn102, leads to UBIAD1 dysfunction and lipid dysregulation. The first is disruption of the cholesterol recognition/interaction amino acid consensus (CRAC) motif in which the p.(Thr103Ile) mutation is located [50]. The CRAC motif is defined as (L/V)X_1–5_-(Y)-X_1–5_-(K/R) and contains three key amino acids: an apolar Leucine (L) or Valine (V) residue, a mandatory central Tyrosine (Y) residue, and a basic Lysine (K) or Arginine (R) residue. In addition to having the three key amino acids, the CRAC motif must be located at the edge of a transmembrane domain or within a transmembrane domain to function properly. Changes to this consensus sequence can lead to cholesterol dysregulation, and it has been shown that ectopic UBIAD1 expression or induction of endogenous UBIAD1 in human cancer cell lines can reduce elevated cholesterol levels [51, 52]. While the p.(Thr103Ile) mutation does not involve one of the three conserved amino acids in the CRAC motif, the motif itself is necessary but not sufficient for optimal cholesterol binding. Analysis of the energy interaction between CRAC motifs and cholesterol shows that nearby amino acids can also play critical roles in modulating cholesterol binding [53, 54]. Given that nearby amino acids can affect cholesterol binding, changes to amino acids within the CRAC motif itself likely also affect binding. Therefore, the p.(Thr103Ile) mutation, while not eliminating the CRAC domain entirely, may damage the ability of UBIAD1 to bind to substrates.

While Lisch et al. concluded that there is no correlation between serum lipid levels and progression of corneal opacifications in SCD, local (i.e., in cornea) cholesterol and lipid metabolism may play a role [6]. This is supported by the finding of significantly elevated cholesterol levels localized to the cornea relative to blood serum levels following intravenous injection of radioactively labeled cholesterol prior to penetrating keratoplasty (PK) [55]. In addition, elevated levels of apolipoproteins A-I, A-II, and E, which are constituents of HDL, have been demonstrated via immunostaining of corneas from individuals with SCD [56]. The fact that the levels of apolipoprotein B, an LDL constituent, are not elevated suggests that a disruption of the HDL metabolic machinery is involved in the pathogenesis of SCD. As the proband that we report had increased serum levels of HDL, but not LDL, one could hypothesize that the p.(Thr103Ile) mutation negatively impacts the binding of UBIAD1 to substrates through interfering with the function of the CRAC domain, disrupting the HDL metabolic pathway within the cornea, and leading to localized cholesterol deposition.

The second potential mechanism via which the p.(Thr103Ile) mutation may lead to UBIAD1 dysfunction and lipid dysregulation involves the disturbance of the wild type transmembrane helix. A number of computational models of the human UBIAD1 protein have been previously published, reporting between seven and ten transmembrane helices in varied locations in the UBIAD1 protein [17, 18, 20, 21, 27, 49, 51]. Although the seven different computational algorithms that we utilized also yielded varied results, each predicted a transmembrane helix in the wild type UBIAD1 protein with a consensus model running from residues 83 to 101. Performing the same modeling with the p.(Thr103Ile) substitution yielded a consensus model with a transmembrane helix running from residues 83 to 103, resulting in localization of the Asn102 and the mutant Ile103 residues within the transmembrane region and disrupting protein function [57]. However, we acknowledge that while computational modeling of protein structure is useful, it is only as reliable as the algorithm on which it is based. Ultimately, any* in silico* analysis should be validated by experimentation. While the structure of a UBIAD1 homologue, AfUbiA from* Archaeoglobus fulgidus*, has been determined via X-ray crystallography, the structure of human UBIAD1 protein has yet to be determined [48]. In order to fully elucidate the functional impact of the mutations associated with SCD, including the novel p.(Thr103Ile) missense mutation that we report, it is necessary to first have comprehensive structural data for the UBIAD1 protein.

In summary, we report the first* de novo* mutation in UBIAD1 associated with Schnyder corneal dystrophy. As the diagnosis of SCD was questioned given the absence of a family history, this case highlights the utility of genetic testing to confirm or refute a presumptive clinical diagnosis. The identified missense p.(Thr103Ile) mutation involves a conserved residue and is predicted to be damaging to the function of the encoded protein, possibly by disrupting the cholesterol recognition/interaction amino acid consensus (CRAC) motif or altering the structure of the transmembrane helix.

## Figures and Tables

**Table 1:** Mutations in *UBIAD1* associated with Schnyder corneal dystrophy.

Exon	Nucleotide change	Amino acid change	Number of families reported	References
1	c.289G>A	p.(Ala97Thr)	1	[18]
1	c.290G>A	p.(Gly98Ser)	1	[19]
1	c.305A>G	p.(Asn102Ser)	20	[15, 17, 18, 20–23]
*1*	*c.308C>T*	*p.(Thr103Ile)*	*1*	*Current study*
1	c.334G>A	p.(Asp112Asn)	1	[18]
1	c.335A>G	p.(Asp112Gly)	1	[17]
1	c.353A>G	p.(Asp118Gly)	1	[21]
1	c.355A>G	p.(Arg119Gly)	1	[17, 20]
1	c.361C>G	p.(Leu121Val)	1	[20]
1	c.361C>T	p.(Leu121Phe)	3	[21, 24]
1	c.365T>A	p.(Val122Glu)	1	[18]
1	c.365T>G	p.(Val122Gly)	1	[18]
1	c.511T>C	p.(Ser171Pro)	1	[21, 25]
1	c.521A>G	p.(Tyr174Cys)	1	[26]
1	c.524C>T	p.(Thr175Ile)	2	[17, 21]
1	c.529G>C	p.(Gly177Arg)	1	[15]
1	c.529G>A	p.(Gly177Arg)	2	[21]
2	c.530G>A	p.(Gly177Glu)	6^*∗*^	[27]
2	c.542A>G	p.(Lys181Arg)	1	[26]
2	c.556G>A	p.(Gly186Arg)	1	[21]
2	c.563T>A	p.(Leu188His)	1	[18]
2	c.695A>G	p.(Asn232Ser)	1	[17]
2	c.697A>C	p.(Asn233His)	1	[26]
2	c.708C>G	p.(Asp236Glu)	1	[21]
2	c.710T>A	p.(Ile245Asn)	1	[23]
2	c.718G>A	p.(Asp240Asn)	1	[28]

^**∗**^Four of the six families may be related and thus may be due to a common founder effect.

**Table 2:** Genotyping for a sex associated marker, amelogenin (AMEL), and 10 short tandem repeats to confirm paternity and maternity of an individual with Schnyder corneal dystrophy.

Allelic marker	Father (I/1)^*∗*^		Child (II/2)^*∗*^		Mother (I/2)^*∗*^		Random match probability
AMEL	Y	X	X	X	X	X	N/A
CSF1PO	11	10	10	10	10	12	0.112
D13S317	11	9	9	12	12	11	0.085
D16S539	11	9	9	11	11	12	0.089
D21S11	32.2	30	30	28	28	30	0.039
D5S818	11	11	11	12	12	12	0.158
D7S820	12	8	8	9	9	12	0.065
TH01	6	6	6	6	6	9	0.081
TPOX	9	10	10	11	11	11	0.195
VWA	14	17	17	18	18	14	0.062

Note: there is greater than 99.999% percent probability that both parents are the biological parents.

^*∗*^IDs in parenthesis refer to pedigree in Figure 1.
